# A metastatic skull tumor from intrahepatic cholangiocarcinoma: A case report and literature review

**DOI:** 10.1097/MD.0000000000018291

**Published:** 2019-12-10

**Authors:** Dawei Wang, Shiwei He, Liang Chu, Qing Chao, Qiujian Zhang, Hansheng Shu

**Affiliations:** a Department of Neurosurgery; b Department of General Surgery, The Second Affiliated Hospital of Bengbu Medical College, Bengbu, Anhui, People's Republic of China.

**Keywords:** craniospinal venous system, intrahepatic cholangiocarcinoma, metastatic skull tumor, occipital bone metastases

## Abstract

**Rationale:**

Intrahepatic cholangiocarcinoma (ICC) originates from the epithelial cells of the secondary branches that are distant from the intrahepatic bile duct. ICC is a rare pathological type of primary liver cancer, with a high malignancy rate and poor prognosis. However, patients with ICC metastasis to the skull are extremely rarely encountered. Herein, we present a case of a metastatic skull tumor from ICC, along with a literature review.

**Patient concerns:**

A 50-year-old right-handed man who did not smoke was diagnosed with a poorly differentiated ICC (T2aN0M0) in segment VI of the liver in February 2017. Hepatectomy was performed. The patient then presented with a painful mass in the posterior occipital region with dizziness experienced since 1 month, for which he underwent posterior occipital craniotomy. Postoperative specimens were sent for pathological examination.

**Diagnoses:**

We diagnosed the patient with a metastatic skull tumor from ICC.

**Interventions:**

The patient underwent posterior occipital craniotomy and total resection of the tumor.

**Outcomes:**

The patient received chemotherapy 1 month after surgery, and after 6 months of follow-up, the patient was alive.

**Lessons:**

ICC often shows metastases to the vertebrae. Therefore, physicians should consider the possibility of metastasis in patients with ICC, especially in those who show a painful skull mass of unknown origin; moreover, among patients with vertebral metastasis, physicians should be very vigilant about an occipital mass. We believe that the craniospinal venous system may be the pathway for occipital metastasis in patients with ICC.

## 1 Introduction

Intrahepatic cholangiocarcinoma (ICC) originates from the epithelial cells of secondary branches that are distant from the intrahepatic bile duct. ICC is a rare pathological type of primary liver cancer, with an incidence of 10% to 15%.^[1]^ However, the incidence of ICC is increasing globally.^[2]^ ICC has a high malignancy rate and poor prognosis.^[3]^ Advanced stage ICC can metastasize to multiple organs, including the lungs, kidneys, and bones.^[3]^ However, patients with ICC metastasis to the skull are extremely rare. Only 4 cases have been reported till date. Therefore, herein, we present a case of a metastatic skull tumor from ICC, along with a literature review. We believe that this summary will be helpful for the diagnosis and treatment of patients with ICC in the future.

## 2 Case report

**Figure 1:**
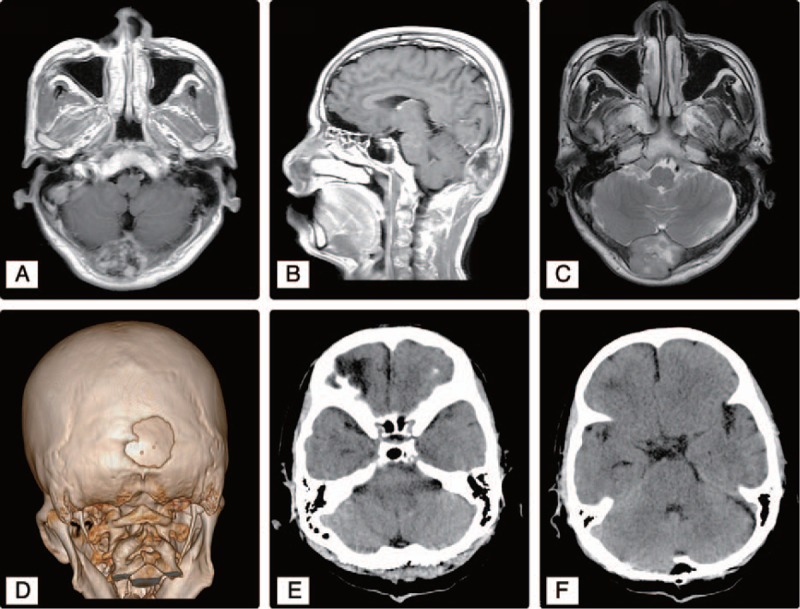
Imaging findings. A–C, Preoperative brain T1 magnetic resonance imaging (sagittal and axial images, respectively) reveals a heterogeneous enhanced mass, measuring approximately 3 cm × 3 cm × 2 cm. The tumor has clear boundaries with the bone tissue, invades the dura mater, compresses the brain tissue, and shows osteolytic changes in the bone. D, Preoperative three-dimensional computed tomography of the skull shows a defect in the bone window of approximately 2.5 cm × 2.5 cm, with invasion to the whole skull layer. E, F, After resection of the mass and surrounding skull, a titanium plate has been used to repair the defect in the skull.

**Figure 2:**
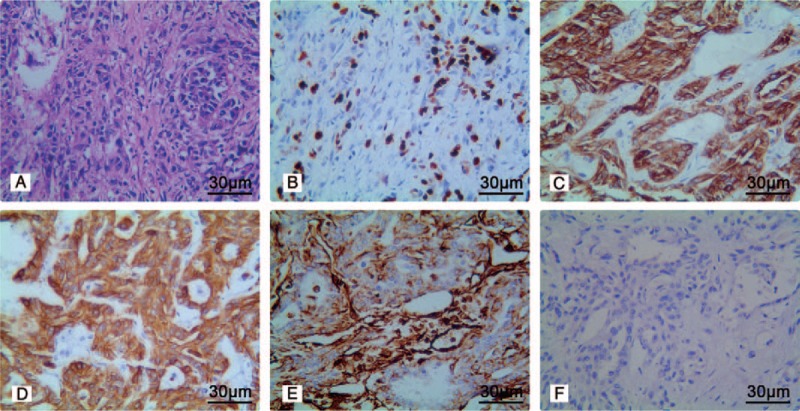
Histopathological staining of the lesion resected during posterior occipital craniotomy. A, Hematoxylin-eosin staining shows a sheet of cells with acinar structures and abundant eosinophils. Individual cells have a large vesicular nucleus (magnification, ×200). B, The Ki-67 labeling index is 70% (magnification, ×200). The tumor shows (C) positive immunoreactivity for CK (magnification, ×200); (D) positive immunoreactivity for CK19 (magnification, 200×); (E) positive immunoreactivity for vimentin (magnification, 200×); and (F) negative immunoreactivity for CK7 (magnification, 200×). CK = cytokeratin.

**Figure 3:**
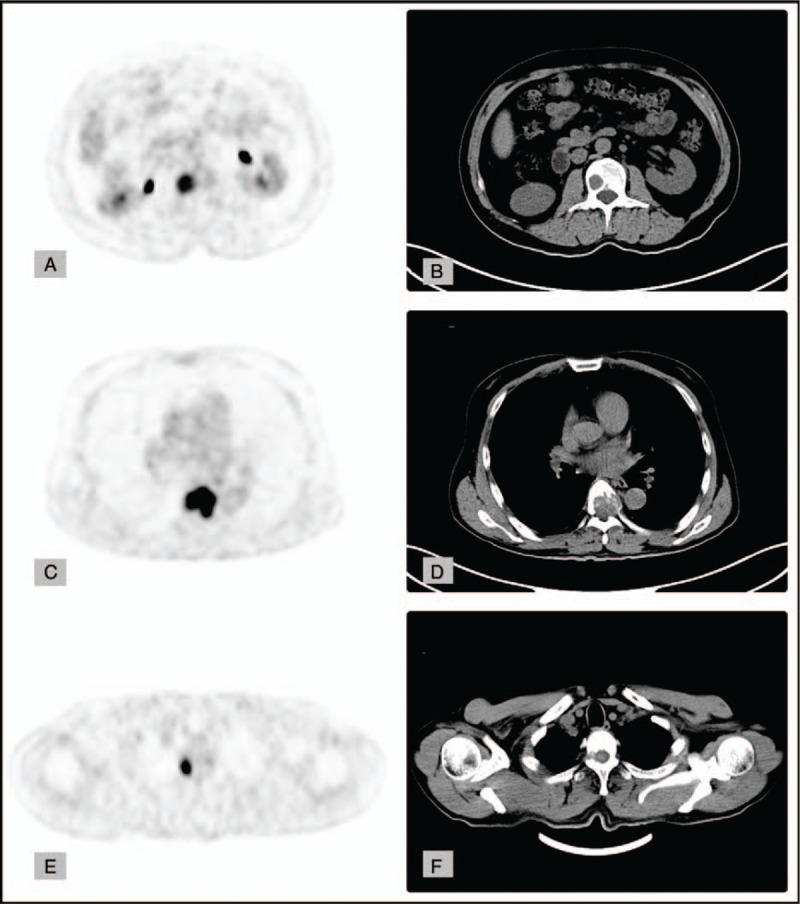
A–F, Positron emission tomography-computed tomography shows vertebral metastasis and increased fluorodeoxyglucose uptake in multiple nodules in the liver and adrenal gland. The location of the metastases is along the lines of the craniospinal venous system.

A 50-year-old right-handed who did not smoke was diagnosed with a 2.2 cm × 2.5 cm poorly differentiated ICC (T2aN0M0) in segment VI of the liver in February 2017. Hepatectomy was performed. The patient had clear resection margins and was treated postoperatively with transcatheter arterial chemoembolization. After perfusion, the patient showed good recovery; after discharge, he had a normal life without any discomfort. However, the patient did not undergo regular follow-up computed tomography (CT) per the doctor's advice, and 1 month later presented with presented with a painful mass. He did not complain of nausea, vomiting, or other neurological deficit symptoms. On physical examination, the tumor was hard, showed poor mobility, was not tender, and had no surface skin ulceration. Brain CT revealed a single mass within the central part of the occipital bone. T1-enhanced brain magnetic resonance imaging (MRI) revealed a heterogeneous enhanced mass, measuring approximately 3 cm × 3 cm × 2 cm. The tumor had clear boundaries along the bone tissue, had invaded the dura mater, compressed the brain tissue, and showed osteolytic changes in the bone. Three-dimensional CT of the skull showed a defect in the bone window of approximately 2.5 cm × 2.5 cm, with invasion to the entire skull layer (Fig. 1). Small pulmonary nodules whose nature was unknown were visible on chest radiographs. The patient underwent posterior occipital craniotomy and total resection of the tumor. The tumor was located under the epicranial aponeurosis and invaded the whole layer of the skull. The lesion in the skull was osteolytic, adhered closely to the dura mater, and was hard to separate; it was closely adherent to the surrounding tissues. The mass was dark red, with a rotten fish-like appearance, and had abundant vascular supply. The mass and the surrounding skull were resected; dural electrocoagulation and titanium plates were used to repair the defect in the skull. Histopathological examination of the specimen showed a sheet of cells with acinar structures and abundant eosinophils. Individual cells had a large vesicular nucleus. Immunohistochemical staining revealed that the tumor was positive for cytokeratin (CK), vimentin, epithelial membrane antigen (EMA), and CK19; and negative for CD34, CD99, Bcl-2, CK7, and thyroid transcription factor *1 (*TTF-1). The Ki-67 labeling index was 70% (Fig. 2). The pathological report suggested a metastatic skull tumor from ICC. Subsequent positron emission tomography (PET)-CT showed increased fluorodeoxyglucose uptake in multiple nodules in the liver and adrenal gland (Fig. 3). The patient received chemotherapy regimen of KNO35 combined with gemcitabine and oxaliplatin 1 month after the surgery, and after 6 months of follow-up, the patient was still alive. The patient did not undergo other treatments or reviews and had no recurrence of the occipital mass.

## 3 Discussion

ICC originates from the epithelial cells of the secondary branches that are distant from the intrahepatic bile duct. ICC is the second most common primary liver cancer, with an incidence of 10% to 15%. ICC is common in patients aged 30 to 50 years, and the incidence of ICC has been increasing worldwide over the last 3 decades. Owing to the low incidence and lack of specific early symptoms of ICC, its diagnosis is challenging; it is usually diagnosed at an advanced stage when surgery is difficult or impossible.^[4,5]^ However, because patients with advanced ICC are prone to recurrence and metastases and have a poor prognosis, the use of therapeutic regimens is limited, with ICC cases showing low sensitivity to chemoradiotherapy. Even after curative resection, the recurrence rates are high and the long-term survival remains poor. The median survival time is only 3 to 6 months.^[1]^ ICC is usually associated with cirrhosis, viral hepatitis B, and hepatitis C.^[6]^ Complete surgical resection is the only possible treatment for patients with ICC.^[7]^

Extrahepatic metastasis of ICC is mainly through the blood and lymph, but most cases of metastasis are to the lungs. Bone metastasis is more common in the spine and ilium, while skull metastasis is very rare. PubMed, Ovid MEDLINE, Ovid EMBASE were searched to identify all published reports addressing skull metastasis of ICC published from inception to September 2019. Search key words included “cholangiocarcinoma,” “intrahepatic cholangiocarcinoma,” “metastatic skull tumor,” “bone metastases,” “skull metastases,” and “treatment, prognosis.” Published conference proceedings were also searched. Till date, only 4 cases of skull metastasis of ICC have been reported in the English literature, including 2 cases of occipital bone metastases (Table 1). The common feature of the current case and the 2 previously reported cases of occipital bone metastases is the coexistence of occipital and vertebral metastases. ^[8,9]^ Herein, we present a case of a metastatic skull tumor from ICC. Of the 4 previously reported cases of intracranial cholangiocarcinoma with skull metastasis, one was detected simultaneously with the primary lesion, and the other 3 were detected after ICC treatment.^[8,9]^

**Table 1:**
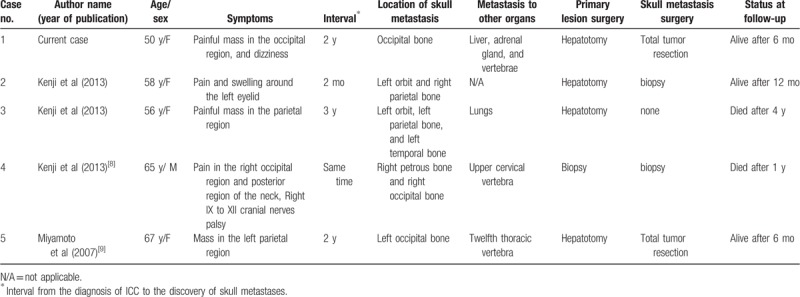
Clinical characteristics of patients with skull metastases from intrahepatic cholangiocarcinoma.

The common features of the 4 previously reported cases and our case were the metastatic tumor invading the skull barrier space as well as the internal and external skull plates, thereby expanding it into a crescent or double convex shape, causing compression and displacement of the local cerebral cortex. The external plate of the skull was eroded, and the tumor invaded the epidural and subdural spaces. Thus, in the previously reported cases, the scalp manifested pain and swelling. The common feature was the double convex shape growth, but the symptoms of intracranial hypertension were not obvious. The reported cases had multiple organ metastases, especially to the spine, which is related to the metastatic route.

There are 2 pathways of bone metastasis from ICC: the hematogenous pathway and the osseous pathway via the craniospinal venous system (CSVS). The CSVS has 2 main systems: 1) the intracranial veins, which include the cortical veins, dural sinuses, cavernous sinuses, and ophthalmic veins; and 2) the vertebral venous system, which includes the vertebral venous plexus (the Batson plexus).^[10]^ The CSVS is characterized by the lack of venous valves, which allow bidirectional blood flow.^[11]^ In the current case, we observed multiple vertebral metastases along the lines of the CSVS. Thus, the CSVS may be one of the metastatic pathways for skull metastasis from ICC.^[8]^ All the previously reported cases were treated with surgery, the pathology confirmed, and the skull metastases removed. This case also chose surgery. The tumor was completely resected. There was no local recurrence at the half-year follow-up, but the prognosis was poor due to multiple metastases.

Extrahepatic metastases of ICC are often observed in the vertebrae. Therefore, physicians should consider the possibility of metastasis in patients with ICC who show a painful skull mass of unknown origin; in particular, among patients with vertebral metastasis, an occipital mass should be considered a possibility. In fact, the CSVS may be the pathway of occipital metastasis from ICC.

## Acknowledgments

The authors thank all the staff of the Neurosurgery Department and General Surgery Department of The Second Affiliated Hospital of Bengbu Medical College for their support.

## Author contributions

**Conceptualization:** Dawei Wang, Shiwei He, Qiujian Zhang, Hansheng Shu.

**Data curation:** Liang Chu, Qiujian Zhang.

**Formal analysis:** Liang Chu.

**Funding acquisition:** Dawei Wang, Shiwei He.

**Software:** Qing Chao.

**Supervision:** Qing Chao.

**Writing – original draft:** Dawei Wang, Shiwei He.

**Writing – review & editing:** Liang Chu.
